# A de novo missense mutation in MPP2 confers an increased risk of Vogt–Koyanagi–Harada disease as shown by trio-based whole-exome sequencing

**DOI:** 10.1038/s41423-023-01088-9

**Published:** 2023-10-12

**Authors:** Xianyang Liu, Jiayu Meng, Xingyun Liao, Yusen Liu, Qian Zhou, Zongren Xu, Shuming Yin, Qingfeng Cao, Guannan Su, Siyuan He, Wanqian Li, Xiaotang Wang, Guoqing Wang, Dali Li, Peizeng Yang, Shengping Hou

**Affiliations:** 1 The First Affiliated Hospital of Chongqing Medical University, Chongqing, China; 2 Chongqing Key Laboratory of Ophthalmology, Chongqing, China; 3 Chongqing Eye Institute, Chongqing, China; 4 Sichuan Provincial Key Laboratory for Human Disease Gene Study, Sichuan Provincial People’s Hospital, University of Electronic Science and Technology of China, Chengdu, 611731 China; 5 Department of Medical Oncology, Chongqing University Cancer Hospital, Chongqing, 400030 China; 6 Shanghai Frontiers Science Center of Genome Editing and Cell Therapy, Shanghai Key Laboratory of Regulatory Biology, School of Life Sciences, East China Normal University, Shanghai, 200241 China; 7 Beijing Institute of Ophthalmology, Beijing Tongren Eye Center, Beijing Tongren Hospital, Capital Medical University, Beijing Ophthalmology & Visual Sciences Key Laboratory, Beijing, 100730 China

**Keywords:** Vogt–Koyanagi–Harada disease, Whole exome sequencing, De novo mutation, Membrane palmitoylated protein 2, Annexin A2, ERK3/IL-17E pathway, Autoimmunity, Immunogenetics, Immunogenetics

## Abstract

Vogt–Koyanagi–Harada (VKH) disease is a leading cause of blindness in young and middle-aged people. However, the etiology of VKH disease remains unclear. Here, we performed the first trio-based whole-exome sequencing study, which enrolled 25 VKH patients and 50 controls, followed by a study of 2081 VKH patients from a Han Chinese population to uncover detrimental mutations. A total of 15 de novo mutations in VKH patients were identified, with one of the most important being the membrane palmitoylated protein 2 (MPP2) p.K315N (MPP2-N315) mutation. The MPP2-N315 mutation was highly deleterious according to bioinformatic predictions. Additionally, this mutation appears rare, being absent from the 1000 Genome Project and Genome Aggregation Database, and it is highly conserved in 10 species, including humans and mice. Subsequent studies showed that pathological phenotypes and retinal vascular leakage were aggravated in MPP2-N315 mutation knock-in or MPP2-N315 adeno-associated virus-treated mice with experimental autoimmune uveitis (EAU). In vitro, we used clustered regularly interspaced short palindromic repeats (CRISPR‒Cas9) gene editing technology to delete intrinsic MPP2 before overexpressing wild-type MPP2 or MPP2-N315. Levels of cytokines, such as IL-1β, IL-17E, and vascular endothelial growth factor A, were increased, and barrier function was destroyed in the MPP2-N315 mutant ARPE19 cells. Mechanistically, the MPP2-N315 mutation had a stronger ability to directly bind to ANXA2 than MPP2-K315, as shown by LC‒MS/MS and Co-IP, and resulted in activation of the ERK3/IL-17E pathway. Overall, our results demonstrated that the MPP2-K315N mutation may increase susceptibility to VKH disease.

## Introduction

Uveitis, which is defined as a severe sight-threatening autoimmune disease, is one of the leading causes of blindness in young and middle-aged people. This condition usually results in irreversible and permanent blindness due to severe damage to the retina and optic nerve as a result of a recurrent inflammatory response. Epidemiological evidence suggests that the rate of blindness caused by uveitis accounts for approximately 25% of all eye diseases in developing countries [1, 2].

Based on pathological features, uveitis can be classified into noninfectious uveitis and infectious uveitis, with Vogt–Koyanagi–Harada (VKH) disease being the most common type of noninfectious uveitis found in Asian, Native American, Hispanic, and Middle Eastern populations [3]. VKH disease is a type of acute, diffuse uveitis with specific systemic symptoms characterized by granulomatous panuveitis, alopecia, vitiligo, hearing loss, and central nervous system abnormalities [4, 5]. All of the known uveitis conditions do not have a well-defined Mendelian pattern of inheritance; some forms are frequently familial [6]. The etiology of VKH disease is complex, with a combination of genetic and autoimmune factors contributing to its development [7–10]. Recent advances in high-throughput sequencing have substantially improved our understanding of the genetic risk factors for VKH disease. Candidate gene association studies and genome-wide association studies of VKH disease have identified several associated loci or genes, such as *IL23R-C1orf141, ADO-ZNF365-EGR2, IL17F*, and *IL23A* [4, 11–13]. However, the genetic etiology of VKH disease remains poorly understood.

Experimental autoimmune uveitis (EAU) can mimic many features of VKH disease and has been extensively used to study the underlying mechanisms that may influence susceptibility to uveitis [14–17]. In this study, C57BL/6 mice were immunized with evolutionarily conserved retinal proteins, e.g., human interphotoreceptor retinoid-binding protein; then, antigen-presenting cells (APCs) were prompted to present antigens to activate T-cell differentiation [18, 19]. Activated T-cell subsets such as pathogenic Th1 and Th17 cells release many inflammatory factors that, in turn, damage the blood–retinal barrier (BRB) and photoreceptor cells, leading to the infiltration and activation of ocular immune cells and the development of uveitis, including VKH disease [20–22]. In particular, retinal pigment epithelium (RPE) cells play an important role in the occurrence and development of uveitis. As a layer of regular polygonal cells, the RPE layer forms the external BRB, limiting exposure of the retina to harmful blood components and maintaining the stability of the retinal microenvironment [23]. In addition, RPE cells can synthesize and secrete a variety of cytokines that nourish and protect photoreceptor cells [24]. Researchers have found that mutations in the RPE65 allele destroy photoreceptor cells and lead to the clinical manifestations of Leber congenital amaurosis, which eventually results in complete blindness [25], suggesting that RPE cells may influence the genetic susceptibility to eye disease.

In this study, to investigate additional genetic variants of VKH disease, we performed trio-based whole-exome sequencing (WES) in 25 VKH trio families, and a de novo mutation, membrane palmitoylated protein 2 (MPP2) p.K315N (c.G945T, NM_001278372), was identified. Furthermore, target region sequencing (TRS) was carried out on the MPP2 gene in 2081 VKH patients. Bioinformatic predictions suggested that the mutation was strongly deleterious and highly conserved in several species. Subsequent studies revealed that the MPP2-N315 mutation exacerbated the inflammatory cascade and destroyed barrier function through the ANXA2/ERK3/IL-17E pathway in EAU and RPE cells. Collectively, our data showed for the first time that the MPP2-K315N mutation may increase genetic susceptibility to VKH disease and provides a novel target for uveitis therapy.

## Materials and methods

### Subjects

All experiments involving humans and mice were approved by the Ethics Committee of the First Affiliated Hospital of Chongqing Medical University (permit numbers: 2009-201008, 2019-296 and 2019-099). The present study adhered to the tenets of the Declaration of Helsinki.

A total of 25 VKH trio families (25 VKH patients and 50 unaffected parents) and 2,081 VKH patients from a Han Chinese population were recruited from the First Affiliated Hospital of Chongqing Medical University, and informed consent was obtained. All VKH patients were registered with the China Human Genetic Resources Management Office (2021CJ2035). All VKH patients enrolled in this study were diagnosed by senior ophthalmologists strictly according to the revised diagnostic criteria 2001 for VKH diseases [26]. If the diagnosis was uncertain, patients with unclear diagnoses were excluded from the present study. Genomic DNA was extracted from peripheral blood using the QIAGEN QIAamp DNA Mini Blood Kit (Hilden, Germany) according to the manufacturer’s recommendations [27].

### Whole-exome sequencing

Whole-exome sequencing was supported by Novogene Technology Co., Ltd. An Agilent SureSelect Human All Exon V6 exome analysis (Agilent, USA) was performed according to the manufacturer’s recommendations [28, 29]. Each capture library was sequenced on the Illumina NovaSeq 6000 system (Illumina, USA) for paired-end 150 bp reads after pooling. The quality assessment tests of raw data were performed using FastQC software (http://www.bioinformatics.babraham.ac.uk/projects/fastqc/), and then, adapter sequences and low-quality fragments were removed from the 3′-end and analyzed by Trim Galore software. The clean sequence fragments were mapped to the human reference genome by BWA software and labeled repeats by Picard tools. The latest Best Practice process of GATK3.4 was used for sample variant detection, and ANNOVAR software was used for gene-based, region-based and filter-based site annotation [30].

### Sanger sequencing

All de novo mutations from WES sequencing were confirmed by reamplification of fragments, and repeat Sanger sequencing was provided by Shanghai Sangon Co., Ltd., in all 25 VKH trio families. Primer sequences are available upon reasonable request. PCR products were sequenced by the Applied BiosystemsTM 3730XL DNA Analyzer (Thermo, USA) according to their recommendations and aligned by ClustalX software.

### Real-time quantitative polymerase chain reaction

RNA was extracted from tissues and cells using TRIzol reagent (Invitrogen, San Diego, California, USA) according to the manufacturer’s instructions [16, 31]. cDNA was synthesized with the RT Master Mix for qPCR Kit (MCE, Shanghai, China). Real-time quantitative PCR was performed by SYBR Green qPCR Master Mix using an ABI 7500 Real-time PCR System (Applied Biosystems, CA, USA) according to the manufacturer’s instructions (MCE, Shanghai, China) [32]. The primers used in this study are shown in Supplementary Table 1. Relative expression was normalized with an internal reference and calculated using the 2^−ΔΔCT^ method.

### Subretinal injection of virus

Sterile injection was performed according to a previously described method [33–35]. In brief, mice were abdominally injected with 1% pentobarbital sodium (50 mg/kg), and tropicamide (0.5%) was dropped on the eyes. Next, an aperture was made using a 30-gauge syringe needle through the upper cornea under a microscope, and then, a 33-gauge blunt needle (Hamilton, CA, USA) was inserted through the aperture into the subretinal space. Finally, 0.5 µl of virus was slowly injected into the space, and the needle was pulled out slowly after a few seconds of pause. Antibiotic ointment was applied to the cornea for three days after this operation.

### EAU induction and assessment

In the present study, 6- to 8-week-old female C57BL/6 J mice were subcutaneously injected with 350 µg of hIRBP651-670 in 0.1 ml of ultra-pure water containing 20% DMSO (Sigma–Aldrich, V900090, USA) emulsified with an equal volume of complete Freund’s adjuvant containing 500 µg of *Mycobacterium tuberculosis* H37R. On the same day, 1 µg pertussis toxin was injected intraperitoneally as previously reported [16, 36]. After 14 days, eyes were examined by slit lamp for clinical scores, and then, they were embedded in paraffin, stained with hematoxylin and eosin (H&E), and scored according to Caspi’s criteria described previously [20, 22].

### Evans blue assay

The breakdown of the blood–retinal barrier (BRB) was evaluated by Evans blue analysis and fluorescein fundus angiography (FFA) on Day 14 after immunization. Mice were injected with 2% Evans blue (Dalian Meilum Biotechnology Co., MB4680, China) through the tail veins and sacrificed two hours later. Eyes were fixed in 4% paraformaldehyde, and then, the retinas were spread with glycerol on the slides and covered under coverslips. Images were collected with an immunofluorescence microscope (Leica, Germany).

### Fluorescein fundus angiography

Mice were injected intraperitoneally with 1% fluorescein and immediately examined in the dark room by fundus microscopes. Sufficient water was given to metabolize the fluorescein after this examination.

### Cell culture

The ARPE19 cell line was purchased from the American Type Culture Collection (ATCC, USA) and cultured in the recommended medium with 10% FBS in an incubator at 37 °C with 5% CO_2_.

### CRISPR/Cas9 gene editing technology

For the generation of the MPP2 gene knockout ARPE19 cell line, the Lonza 4D Nucleofector Kit (V4XP-3032) was used for nucleofection according to the manufacturer’s recommendations. In brief, Cas9 protein was purchased from Aldveron, and two sgRNAs (SgRNA1: 5′GATCCCTCCCCAGTGCCACG3′; SgRNA2: 5′AAGTCCCATAGTAAGATCCC3′) targeting the MPP2 gene were designed using Benchling software (http://www.benchling.com) and then synthesized from Synthego. ARPE19 cells were nucleofected with a ribonucleoprotein (RNP) complex (100 pmol Cas9 protein and 300 pmol sgRNA) using a 4D nucleofector system (Lonza) and the DN-100 program. After electroporation, the cells were washed once using 1 × PBS buffer and then cultured in an incubator at 37 °C and 5% CO_2_. Two days after electroporation, genomic DNA was extracted from several cells using the Genome Extraction Kit (Tiangen, DP304). For determination of the knockout efficiency of the two sgRNAs, the targeted region was amplified using TransStart Fast Pfu DNA polymerase (TransGene Biotech) and sent for Sanger sequencing (forward primer: 5′AGGCCAAGGAAATTGTCCCC3′; reverse primer: 5′CTCCACAAGCTGGCAGTCAA3′). The knockout efficiency was analyzed and quantified from Sanger sequencing data using Inference of CRISPR Edits (ICE) software (http://ice.synthego.com).

### Lentiviral cell transduction

Lentiviruses targeting MPP2 and ANXA2 were used to mutate or knockdown the expression of these genes, respectively. The vehicle or scramble was used to produce control lentiviruses. Briefly, the expression vectors and packaging vectors were transfected into ARPE19 cells using polybrene to generate a stably expressing strain according to the manufacturer’s instructions.

### Cell Counting Kit-8 assay

ARPE19 cells were seeded into 96-well plates at a density of 5 × 10^3^ cells/well and cultured for 24 h. The culture medium was replaced on Days 2 and 4. After three washes with PBS, 10 μl of Cell Counting Kit-8 **(**CCK-8) reagent (C0005, TargetMol, USA) was added to each well, and the cells were incubated at 37 °C for another 2 h. The absorbance was measured at an optical density (OD) of 450 nm using a microplate reader (Thermo Fisher Scientific, Inc., MA, USA).

### Enzyme-linked immunosorbent assay

Enzyme-linked immunosorbent assays (ELISAs) were used to determine the secretion of the studied proteins. Supernatant of ARPE19 cells was used to detect IL-1β, IL-10, IL-17E and VEGFA secretion. Retinal homogenate was used to detect the release of IL-17E. The experiment was performed according to the manufacturer’s instructions. The absorbance of the samples was measured with a microplate spectrophotometer at 450 nm. The obtained values were recalculated to the total protein level.

### Transepithelial electrical resistance assay

ARPE19 cells were digested and cultured in Transwell plates until a tight monolayer was formed. Transepithelial electrical resistance **(**TEER) was measured using a voltmeter: TEER (ohmic per square centimeter) = (total resistance-blank resistance) (ohmic) × area (square centimeter).

### Phenol red infiltration assay

Phenol red leakage was also employed to examine the integrity of the ARPE19 cell layers. Hank’s balanced salt solution (HBSS) was used to rinse cells on electrospun PLA nanofiber scaffolds three times. The Transwell models were then filled with HBSS to pre-equilibrate. The apical side was then filled with phenol red solution (0.62 mg/L) instead of HBSS. Following incubation, the solutions on both sides were collected, and then, the phenol red concentration was detected with a microplate spectrophotometer at 550 nm.

### Transmission electron microscopy

The tight junctions of ARPE19 cells were observed by transmission electron microscopy (TEM). In brief, ARPE19 cells were fixed with 2.5% glutaraldehyde at 25 °C. Then, the cells were dehydrated with graded acetone (50, 70, 90, and 100%) and embedded in Epon 812 resin (Head, Beijing, China) overnight. Ultrathin sections (50–70 nm) were acquired using an EM UC7 ultramicrotome (Leica, Germany) and then stained with 2% sodium acetate lead citrate. Finally, the sections were observed under an FEI Tecnai TEM (Thermo Fisher Scientific, Waltham, MA, USA).

### Immunofluorescence assay

ARPE19 cells were seeded on climbing slices for 24 h. After three washes with PBS, the cells were fixed in 4% paraformaldehyde (PFA) for approximately 20 min and then permeabilized with 0.5% Triton X-100 for 10 min. The primary antibody against ZO-1 (ab221547, Abcam, UK) was incubated at 4 °C overnight and then incubated with the Cy3 fluorescent-conjugated secondary antibody (Beyotime, Shanghai, China) at room temperature for 1 h. Images were captured by fluorescence microscopy (SP8; Leica).

### Western blotting

The retinas and RPE cells were lysed with radioimmunoprecipitation assay (RIPA) lysis buffer (Beyotime, Shanghai, China) containing 1% protease inhibitor (Beyotime, Shanghai, China), and the protein concentration was measured using a bicinchoninic acid assay kit (Beyotime, Shanghai, China). The protein was separated and electroblotted onto polyvinylidene difluoride membranes. Then, the membranes were blocked in 5% nonfat milk and incubated with primary antibodies. Finally, bands were visualized using the Mengbio kit and quantified by ImageJ. The primary antibodies used in this study are shown in Supplementary Table 2.

### Tandem mass tag-based quantitative proteomics

The retinas of MPP2-N315 and MPP2-K315 mutant mice with EAU were lysed in 6 M guanidine hydrochloride, homogenized with a homogenizer (MP Biomedicals, Irvine, CA, USA), sonicated, and then boiled for 10 min, as well as ARPE19 cells with MPP2-N315 mutation or wild-type. After centrifugation, the supernatants were processed for filter-aided sample preparation (FASP) digestion using trypsin, and then, 100 μg of the peptide mixture from each sample was labeled using tandem mass tag (TMT) 10plex Isobaric Label Reagent (Thermo Fisher Scientific, 90110) according to the manufacturer’s instructions. The TMT-labeled digested samples were separated into 15 fractions using the Pierce High pH Reversed-Phase Peptide Fractionation Kit (Thermo Fisher Scientific, 84868). Each fraction was separated with an EASY-nLC 1000 Liquid Chromatograph (Thermo Fisher Scientific) and then subjected to Liquid Chromatograph Mass Spectrometer (LC‒MS/MS) analysis on a Q Exactive HF-X Hybrid Quadrupole-Orbitrap Mass Spectrometer (Thermo Fisher Scientific) for 90 min. The spectra were analyzed by Proteome Discoverer (Thermo Fisher Scientific, version 1.4) and then subjected to a database search using the MASCOT search engine (Matrix Science, Boston, MA, USA; version 2.2) for peptide identification. All identified proteins were determined using a false discovery rate (FDR) threshold of <0.01. After retrieval from the UniProtKB database and searching against the SwissProt database (mouse), the top BLAST hits were processed for GO enrichment analysis using Blast2GO (BioBam, Valencia, Spain; version 3.3.5) [37].

### Liquid chromatograph mass spectrometer analysis

For identification of potential MPP2-binding proteins, ARPE19 cells transduced with wild-type or MPP2-N315 mutant were treated with MG132 (10 μm) for 8 h before being collected for assays. MPP2 was pulled down by IP using anti-MPP2 antibody and protein G agarose (ROCHE, 11719416001) at 4 °C. Liquid chromatography‒mass spectrometry (LC‒MS/MS) analysis was performed at Shanghai Applied Protein Technology [38].

### Coimmunoprecipitation assay

The lysate was added to the cell culture plate for full cell lysis at 4 °C. The lysate was centrifuged at 12,000 rpm for 10 min, and the supernatant was collected and incubated with anti-MPP2 antibody at 4 °C overnight. After binding with protein A/G Sepharose beads (ab206996, Abcam, UK), the protein was eluted with SDS loading buffer and detected by western blotting.

### Statistical analysis

Data were analyzed by SPSS software 20.0 (IBM, USA), and figures were made using Prism version 8.0 (GraphPad, San Diego, USA). Statistical differences were defined as *P* <  0.05.

## Results

### Identification of the de novo mutation MPP2-K315N in trio-family–based analysis of VKH patients

**Fig. 1:**
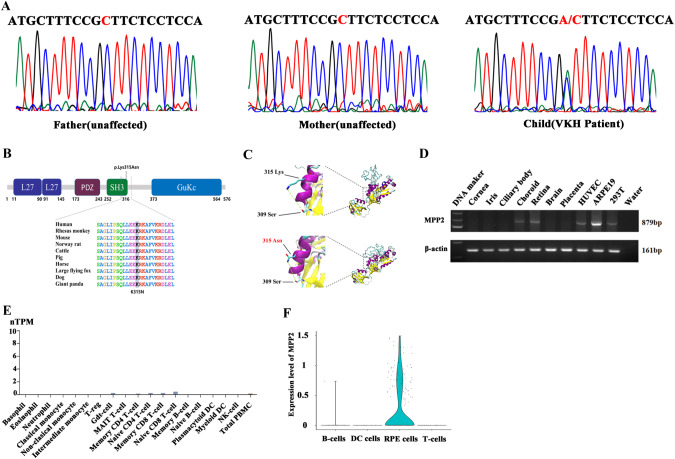
Identification of de novo mutations for VKH disease. **A** Identification of the de novo MPP2 p.K315N mutation in one VKH trio family. **B** The predicted conservation for the MPP2-K315N mutation in humans, Rhesus monkeys, mice, etc. **C** The structure prediction of the MPP2-K315 and MPP2-N315 proteins. **D** The expression profile of the MPP2 gene in several human tissues and cell lines. **E** The nTPM of MPP2 in immune cells. **F** The expression levels of MPP2 in B cells, DC cells, RPE cells and T cells

To investigate the genetic etiology of VKH disease, we performed a WES study enrolling 25 VKH patients (average age, 16.4 years; age range, 7–24 years) and 50 unaffected parents from the Han Chinese population. A total of 15 de novo mutations were identified and confirmed by Sanger sequencing (Table 1). Among them, the c.G945T: p.K315N mutation in MPP2 (exon 9, NM_001278372) was found in one trio family (Fig. 1A). Then, we examined the expression of MPP2 in VKH patients with and without the MPP2-K315N mutation. RT‒qPCR results confirmed no significant difference between them, suggesting that the de novo mutation MPP2-K315N did not affect MPP2 expression (Supplementary Fig. 1). Notably, this mutation was found for the first time in VKH disease in this study and is not present in recognized datasets, including the GWAS Catalog population, 1000 Genome Project (1000 g) Chinese population, 1000 g East Asian population, 1000g_ALL, esp6500si_all (NHLBI-ESP project), Genome Aggregation Database (GnomAD) East Asian population, and GnomAD_ALL population. In addition, the MPP2-K315N mutation was predicted to be deleterious by bioinformatics analysis software. The Sorting Intolerant From Tolerant (SIFT) score was 0.032, indicating that the mutation may have a detrimental effect on protein function. The Polymorphism Phenotyping v2 (Polyphen2) HVAR and Polyphen2_HDIV scores were 0.988 and 0.999, respectively, suggesting that the mutation is likely to be damaging. The MutationTaster and MutationAssessor scores were 0.999 and 2.615, respectively, indicating that the mutation is likely to be disease causing and may cause moderate changes in protein function. The Functional Analysis Through Hidden Markov Models (FATHMM) score was -1.590, indicating that the mutation is likely to be deleterious (Table 2). Taken together, the above results showed that the MPP2-K315N mutation is a deleterious or disease-causing mutation.

**Table 1:** The Summary of 15 de novo mutations in 25 VKH trios families

GeneName	Chr	Pos	Ref	Alt	AAChange	gerp + +gt2
AXDND1	1	179497570	T	G	c.2715+4 T > G	0
ADAMTS3	4	73186589	T	C	c.946-2 A > G	6.07
TAS2R5	7	141490904	C	A	c.C743A:p.P248H	3.37
CA9	9	35675792	C	A	c.C468A:p.C156X	2.24
SIK3	11	116728777	G	A	c.C3080T:p.P1027L	3.64
PTPN11	12	112924295	C	T	c.C1253T:p.T418M	4.26
ARHGAP5	14	32561439	C	G	c.C1564G:p.L522V	0
RYR3	15	33893642	T	C	c.T1811C:p.L604P	4.68
RPAP1	15	41823223	G	A	c.C941T:p.P314L	5.36
HAS3	16	69143434	G	A	c.G136A:p.G46S	5.6
MPP2	17	41958658	C	A	c.G945T:p.K315N	0
KCTD1	18	24056619	C	T	c.G169A:p.G57R	5.63
ZNF236	18	74607146	A	G	c.A1595G:p.K532R	5.48
DNMT3L	21	45670810	G	A	c.C792T:p.H264H	2.87
PLXNA3	X	153695441	C	T	c.C3149T:p.T1050M	5.8

*Chr* Chromosome, *Ref* Reference, *Alt* Alternative, *AAChange* Amino Acid Change, *gerp* genomic evolutionary rate profiling

**Table 2:** The deleteriousness of MPP2-K315N mutation by predicted software

MPP2	SIFT	Polyphen2_HVAR	Polyphen2_HDIV	MutationTaster	MutationAssessor	FATHMM
K315N	0.032, D	0.988, PD	0.999, PD	0.999, DC	2.615, M	−1.590, D

*SIFT* Sorting Intolerant From Tolerant, *Polyphen2* Polymorphism Phenotyping v2, *FATHMM* Functional Analysis Through Hidden Markov Models, *D* deleterious, *PD* Probably damaging, *DC* Disease causing, *M* Medium

Additionally, the MPP2-K315N mutation was located in highly conserved amino acids across different species, including humans, rhesus monkeys, mice, Norway rats, cattle, pigs, horses, large flying foxes, dogs, and giant pandas (Fig. 1B). The functional protein structure of the MPP2-K315N mutation was predicted using SWISS-MODEL (https://swissmodel.expasy.org/), which revealed that the polar interaction between lysine (Lys) and serine (Ser) is stronger than that between asparagine (Asn) and serine (Fig. 1C), suggesting its substantial influence on the structure or function of the MPP2 protein. To further explore the relationship between MPP2 mutations and VKH disease, we performed TRS on the MPP2 gene in 2081 VKH patients and revealed another 24 novel mutations in the exonic region of the MPP2 gene (Table 3). However, we did not find the MPP2-K315N mutation in these VKH patients, suggesting that this is a rare and novel variant in the population. The expression of MPP2 was examined in various human tissues and cultured human cell lines. The results showed that choroid, retina, adult retinal pigment epithelial cell line 19 (ARPE19) cells, human umbilical vein endothelial cells (HUVECs), and human embryonic kidney 293 T (HEK293T) cells expressed MPP2, with expression levels in ARPE19 being the highest (Fig. 1D). We also analyzed the expression of MPP2 in immune cells using the Human Protein Atlas website (https://www.proteinatlas.org/ENSG00000108852-MPP2/immune+cell) and found very low expression of MPP2 in immune cells, including monocytes, T cells, B cells, DC cells, and NK cells (Fig. 1E). Moreover, the expression of MPP2 was examined by analyzing the fetal retinal scRNA-seq data from our previous study [39], which also showed that MPP2 is mainly expressed in RPE cells but barely expressed in T cells, B cells, and DC cells (Fig. 1F). Collectively, our results suggested that the de novo mutation MPP2-K315N is associated with VKH disease and that this mutation may play a crucial role in this disease by regulating the function of RPE cells.

**Table 3:** The MPP2 mutations in 2081 VKH patients by target region sequencing (TRS)

AAChange	cDNAchange	exon	Mutations(frequency)	Gene	ExonicFunc
A5T	G13A	exon1	5/4162	NM_001278372	nonsynonymous SNV
S15C	C44G	exon1	25/4162	NM_001278376	nonsynonymous SNV
E26A	A77C	exon1	2/4162	NM_001278370	nonsynonymous SNV
W30X	G89A	exon1	26/4162	NM_001278370	stopgain SNV
W30X	G90A	exon1	1/4162	NM_001278370	stopgain SNV
L41F	C121T	exon2	1/4162	NM_001278370	nonsynonymous SNV
L41P	T122C	exon2	1/4162	NM_001278370	nonsynonymous SNV
N61I	A182T	exon4	1/4162	NM_001278372	nonsynonymous SNV
T82M	C245T	exon5	1/4162	NM_001278372	nonsynonymous SNV
P158L	C473T	exon6	1/4162	NM_001278372	nonsynonymous SNV
G172R	G514A	exon6	1/4162	NM_001278372	nonsynonymous SNV
R222H	G665A	exon7	1/4162	NM_001278372	nonsynonymous SNV
P246S	C736T	exon7	1/4162	NM_001278372	nonsynonymous SNV
H247Y	C739T	exon7	1/4162	NM_001278372	nonsynonymous SNV
K271E	A811G	exon8	1/4162	NM_001278372	nonsynonymous SNV
G335S	G1003A	exon10	1/4162	NM_001278372	nonsynonymous SNV
R387Q	G1160A	exon11	2/4162	NM_001278372	nonsynonymous SNV
T409A	A1225G	exon12	1/4162	NM_001278372	nonsynonymous SNV
Y423S	A1268C	exon12	1/4162	NM_001278372	nonsynonymous SNV
N473S	A1418G	exon12	1/4162	NM_001278372	nonsynonymous SNV
A507V	C1520T	exon13	1/4162	NM_001278372	nonsynonymous SNV
R524Q	G1571A	exon14	1/4162	NM_001278372	nonsynonymous SNV
E527G	A1580G	exon14	1/4162	NM_001278372	nonsynonymous SNV
P567L	C1700T	exon14	1/4162	NM_001278372	nonsynonymous SNV

### MPP2-N315 mutation exacerbates EAU inflammation and BRB breakdown

For determination of the effects of the MPP2-K315N mutation on EAU inflammation, recombinant MPP2-K315 WT and MPP2-N315 mutant adeno-associated viruses (AAVs) with EGFP tags were constructed and used to infect mice by subretinal injection (Supplementary Fig. 2A, B). The efficiency of MPP2 overexpression in the retina was high, and the duration of overexpression was long (Supplementary Fig. 2C). In addition, we examined whether other tissues of mice expressed the MPP2 mutation; ultimately, the RT‒PCR results showed that only the injected retina specifically expressed the EGFP-tag of MPP2 mutant AAVs (Supplementary Fig. 2D). After subretinal injection of the AAVs, we observed anterior segment- and fundus-related VKH-like symptoms, such as ocular inflammatory phenotypes and BRB leakage, but no skin damage, white hair or blindness in the MPP2-N315 AAV-treated mice without EAU (Supplementary Fig. 2E–J).

**Fig. 2:**
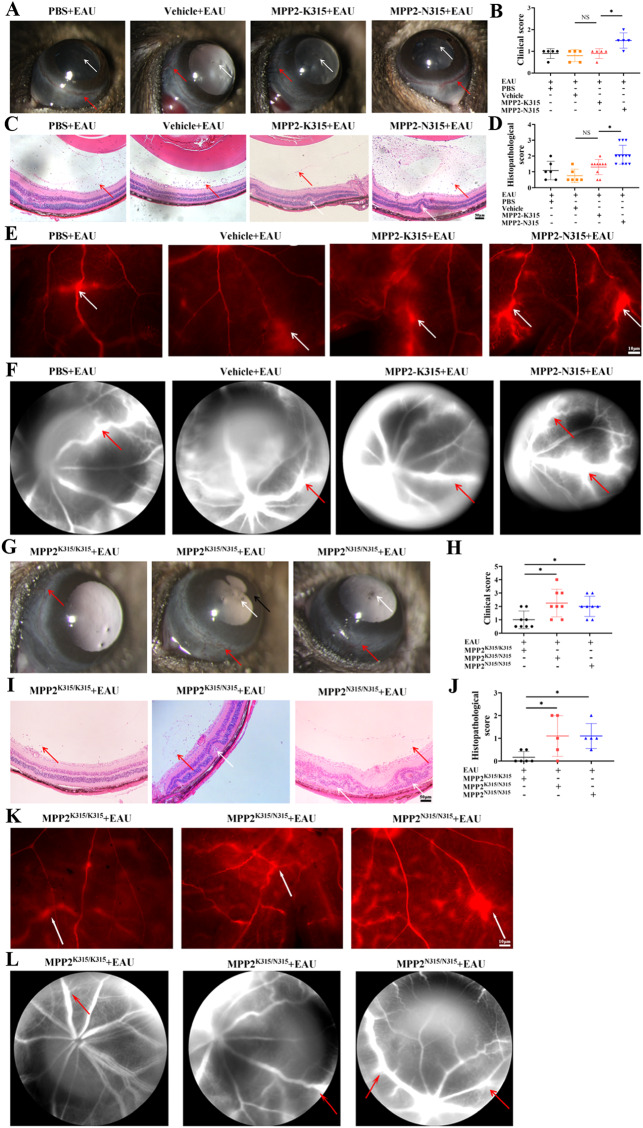
Aggravation of inflammatory response and destruction of the BRB in MPP2-N315 mutant mice with EAU. **A**, **B** Anterior segment inflammation and clinical scores of mice with EAU treated with PBS, vehicle, MPP2 wild-type or MPP2-N315 mutant AAV. White arrow, inflammatory cells. Red arrow, conjunctival and ciliary hyperemia (*n* = 5/group; mean ± SD; NS *p* > 0.05, *p < 0.05; Mann–Whitney U test). **C**, **D** Hematoxylin-eosin staining and pathological scores of immunized mice with PBS, vehicle, MPP2 wild-type or MPP2-N315 mutant AAV. Red arrow, inflammatory cells. White arrow, retina fold (scale bar, 50 μm) (*n* = 6–12/group; mean ± SD; NS *p* > 0.05, *p < 0.05, **p < 0.01; Mann–Whitney U test). **E** Representative retinal flat-mount images of Evans blue on Day 14 after immunization in the four groups mentioned above. White arrow, vessel leakage (scale bar, 50 μm). **F** Representative images of fluorescein fundus angiography in the above four groups. Red arrow, vessel leakage. **G**, **H** Anterior segment inflammation and clinical scores of EAU in knock-in mice with MPP2-K315 or heterozygous or homozygous MPP2-N315 mutation. White arrow, inflammatory cells. Red arrow, conjunctival and ciliary hyperemia. Black arrow, posterior synechiae (*n* = 8/group; mean ± SD; *p < 0.05; Mann–Whitney U test). **I**, **J** Hematoxylin-eosin staining and histopathological scores in MPP2-K315 or heterozygous or homozygous MPP2-N315 knock-in mice with EAU. Red arrow, inflammatory cells. White arrow, retina fold (scale bar, 50 μm) (*n* = 5–6/group; mean ± SD; *p < 0.05; Mann–Whitney U test). **K** Images of Evans blue assays in the three groups mentioned above. White arrow, vessel leakage (scale bar, 10 μm). **L** Pictures of fluorescein fundus angiography in the three groups. Red arrow, vessel leakage

Subsequently, mice were infected with PBS, vehicle, MPP2-K315, or MPP2-N315 mutant AAVs, and they were used to establish an EAU model. We found that the clinical scores of the mice with EAU treated with MPP2-K315 AAV were not significantly different than those of the mice treated with PBS or vehicle on Day 14; in contrast, the clinical scores of the mice with EAU treated with MPP2-N315 AAV were significantly higher than those of the mice treated with MPP2-K315 AAV (Fig. 2A, B). A comparison of the histopathological scores also showed a similar trend (Fig. 2C, D). In addition, vascular staining and fluorescein fundus angiography revealed that the BRB was damaged and that the leakage of retinal vessels was exacerbated in the MPP2-N315 AAV-treated mice with EAU on Day 14 (Fig. 2E, F). Furthermore, to confirm the effect of the MPP2-N315 mutation on EAU phenotypes, we constructed knock-in mice with the MPP2-N315 (c. G873T, NM_016695.4) mutation, which corresponds to the human MPP2-N315 mutation (c.G945T). MPP2-N315 knock-in mice are genetically engineered mice that have a specific gene altered in their genome, and the endogenous MPP2 WT allele of knock-in mice was completely replaced with a mutant allele. Similarly, the clinical and histopathological manifestations were significantly worsened in the homozygous or heterozygous MPP2-N315 knock-in mice compared to the MPP2-K315 knock-in mice after EAU induction on Day 14 (Fig. 2G–J). Retinal vascular leakage and BRB destruction were also exacerbated in the homozygous or heterozygous MPP2-N315 knock-in mice with EAU on Day 14 (Fig. 2K, L). To investigate the time course of clinical symptoms in the MPP2-N315 mutant mice after EAU, we observed anterior segment and fundus images on Days 8, 11, 14, 17, and 20. Both the clinical and pathological scores showed that the inflammatory response was aggravated with prolonged time, peaking on the 14th day and then gradually subsiding thereafter (Supplementary Fig. 2K–N). Additionally, we compared the protein level of MPP2 between the MPP2-K315 and MPP2-N315 knock-in mice, but western blotting results showed no significant difference (Supplementary Fig. 2O, P).

In summary, these results showed that the MPP2-N315 mutation is highly pathogenic and exacerbates EAU manifestation but does not affect MPP2 expression.

### The MPP2-N315 mutation promotes the secretion of inflammatory factors and impairs barrier function in RPE cells

**Fig. 3:**
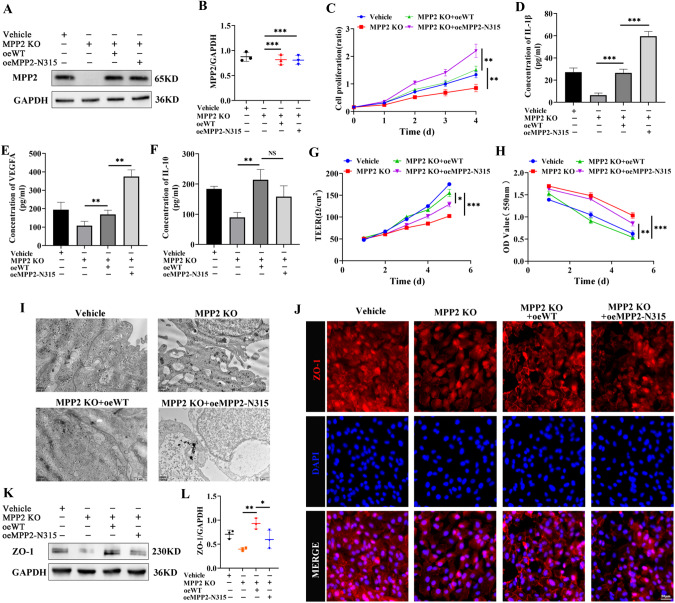
The effects of MPP2 mutation in ARPE19 cells with MPP2 deletion. **A**, **B** The KO efficiency of MPP2 and overexpression level of MPP2 wild-type or the MPP2 mutant (*n* = 3/group; mean ± SD; ***p < 0.001; one-way ANOVA). **C** The proliferation of ARPE19 cells with vehicle, MPP2 KO or overexpression of MPP2 mutant lentiviruses after MPP2 deletion (*n* = 5/group; mean ± SD; **p < 0.01; one-way ANOVA). **D**–**F** The secretion levels of inflammatory factors including IL-1β, VEGFA and IL-10 by ELISA kits (*n* = 6/group; mean ± SD; NS > 0.05, **p < 0.01, ***p < 0.001; one-way ANOVA). **G** The electrical resistance of ARPE19 cells with vehicle, MPP2 KO or overexpression of MPP2 mutant lentiviruses after MPP2 deletion (*n* = 3/group; mean ± SD; *p < 0.05, ***p < 0.001; one-way ANOVA). **H** The phenol red leakage of ARPE19 cells with vehicle, MPP2 KO or overexpression of MPP2 mutant lentiviruses after MPP2 KO (*n* = 5/group; mean ± SD; **p < 0.01, ***p < 0.001; one-way ANOVA). **I** Images of transmission electron microscope (TEM) in the four groups mentioned above (scale bar, 1 μm). **J** The immunofluorescence results of ZO-1 (scale bar, 50 μm). **K**, **L** The protein expression and quantification of ZO-1 (*n* = 3/group; mean ± SD; *p < 0.05, **p < 0.01; one-way ANOVA)

RPE cells play a crucial role in maintaining retinal microenvironmental homeostasis and protecting photoreceptor cells. Structural or functional impairment of RPE cells can lead to a variety of retinopathies [40, 41]. To investigate the effect of the MPP2-K315N mutation on RPE cells, we employed CRISPR‒Cas9 gene editing technology to delete intrinsic MPP2. Sanger sequencing of the genomic DNA of the Cas9-treated ARPE19 cells validated the successful generation of MPP2 knockout cells with a 64 bp deletion (Supplementary Fig. 3A). According to the Inference of CRISPR Edits (ICE) software (http://ice.synthego.com) analysis [42], we observed that the main gene editing outcomes were frameshift mutations accounting for 99%, verifying that the MPP2 gene was successfully knocked out in ARPE19 cells (Supplementary Fig. 3B). We then produced MPP2 knockout (KO) ARPE19 cells overexpressing WT or the MPP2-N315 mutation. Western blotting revealed that the overexpression of WT or the MPP2-N315 mutation reached the vehicle level (Fig. 3A, B). MPP2, as a member of a family of membrane-associated guanylate kinase homologs, regulates cell proliferation, signaling pathways, and intracellular junctions [43]. We therefore explored the effect of MPP2 mutation on cell proliferation. The CCK-8 assay indicated that cell proliferation was significantly reduced in the ARPE19 cells with MPP2 KO and could be rescued by MPP2 overexpression. MPP2 KO cells with oeMPP2-N315 had greater proliferation than those with oeWT (Fig. 3C). Excessive proliferation of RPE cells caused by the MPP2-N315 mutation might synthesize and secrete more inflammatory cytokines, leading to choroidal neovascularization, retinal impairment, and even blindness. Additionally, uncontrolled multiplication of RPE cells may lead to the development of proliferative vitreoretinopathy, resulting in visual impairment and retinal damage [44].

Previous studies have demonstrated that IL-1β in RPE cells promotes inflammation [45] and that VEGFA exacerbates retinal vessel permeability and leakage [46]; however, IL-10 inhibits the inflammatory response [47]. Consequently, the secretion levels of IL-1β, VEGFA, and IL-10 in ARPE19 cells were measured using the corresponding ELISA kits. We found that the levels of these cytokines were all increased in the MPP2 KO ARPE19 cells with oeWT compared to the MPP2 KO ARPE19 cells. Moreover, an overexpressed MPP2-N315 mutation in MPP2 KO ARPE19 cells increased the levels of IL-1β and VEGFA (Fig. 3D–F), suggesting that the MPP2-N315 mutation aggravated the inflammatory response in ARPE19 cells. Additionally, to determine the role of the MPP2-N315 mutation in the barrier function of ARPE19 cells, we performed a transepithelial electrical resistance (TEER) assay and phenol red infiltration test. The results indicated that the barrier function of the MPP2 KO cells with oeMPP2-N315 was impaired compared to that of those with oeWT (Fig. 3G, H). To further confirm the effect of the MPP2-N315 mutation on the barrier function of RPE cells, we performed transmission electron microscopy (TEM) analysis, immunofluorescence, and western blotting. TEM showed that cell tight junctions were worsened in the oeMPP2-N315 ARPE19 cells after intrinsic MPP2 deletion (Fig. 3I). Similarly, immunofluorescence revealed that the expression of zona occludens-1 (ZO-1) was reduced and cell tight junctions were impaired in the MPP2 KO cells with the oeMPP2-N315 mutation (Fig. 3J), and these results were further confirmed by western blotting (Fig. 3K, L). Taken together, these results suggested that the MPP2-N315 mutation promoted inflammatory development and damaged the barrier function in RPE cells, which mediated the key pathogenicity of RPE cells in VKH disease.

### The MPP2-N315 mutation aggravates inflammation through the ERK3/IL-17E pathway in vivo and in vitro

**Fig. 4:**
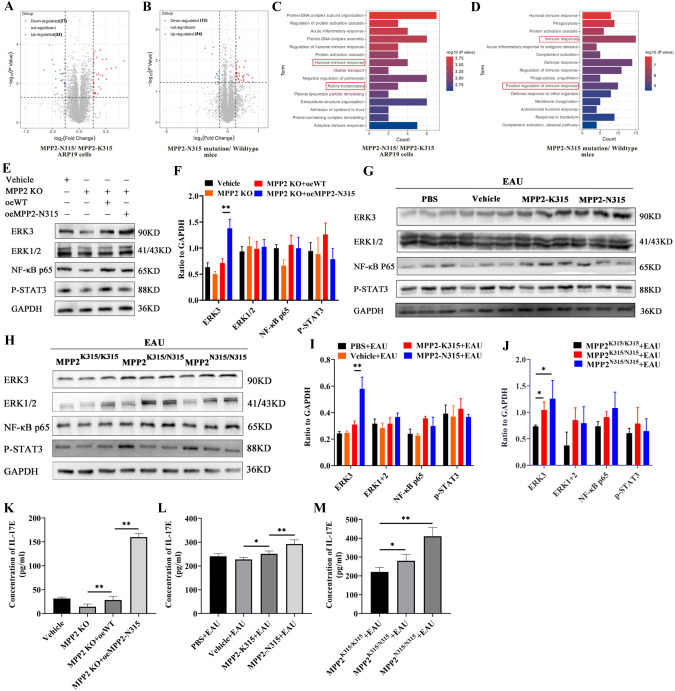
Increased expression of inflammatory-related proteins after MPP2-N315 mutation. **A** The volcanic map of DEGs in MPP2-K315 or MPP2-N315 mutant ARPE19 cells. **B** The volcanic map of DEGs in MPP2 wild-type or mutant mice with EAU. **C**, **D** GO enrichment analysis of proteomics in vitro and in vivo. **E**, **F** Protein levels and quantitative charts of ERK3, ERK1/2, NF-κB p65, and p-STAT3 in vitro (*n* = 3/group; mean ± SD; **p < 0.01; one-way ANOVA). **G**, **I** Protein expression and quantification of ERK3, ERK1/2, NF-κB p65 and p-STAT3 in PBS, vehicle, MPP2-K315 or MPP2-N315 AAV-administered mice with EAU modeling (*n* = 3/group; mean ± SD; ** *p* < 0.01; one-way ANOVA). **H**, **J** Protein levels and quantitative graphs of ERK3, ERK1/2, NF-κB p65 and p-STAT3 in MPP2-K315 or homozygous or heterozygous MPP2-N315 knock-in mice with immunization (*n* = 3/group; mean ± SD; *p < 0.05; one-way ANOVA). **K** The extracellular protein level of IL-17E (*n* = 6/group; mean ± SD; **p < 0.01; one-way ANOVA). **L** The secretion level of IL-17E in mice with EAU treated with PBS, vehicle, MPP2-K315 or MPP2-N315 AAV (*n* = 6/group; mean ± SD; *p < 0.05, **p < 0.01; one-way ANOVA). **M** The secretion of IL-17E in MPP2-K315 or homozygous or heterozygous MPP2-N315 knock-in mice with immunization (*n* = 6/group; mean ± SD; *p < 0.05, **p < 0.01; one-way ANOVA)

To investigate the underlying mechanism of MPP2 mutation in EAU, we performed tandem mass tag (TMT) proteomics in MPP2 mutant ARPE19 cells and MPP2 mutant mice with EAU. A total of 6007 proteins were identified in ARPE19 cells with the MPP2-N315 mutation, including 33 upregulated proteins and 27 downregulated proteins (Fig. 4A). In addition, a total of 6539 proteins were identified in the MPP2-N315 mutant mice with EAU, of which 34 were significantly upregulated and 12 were significantly downregulated (Fig. 4B). In particular, GO analysis revealed that the MPP2-K315N mutation was associated with the immune response and retinal homeostasis (Fig. 4C, D). We therefore examined several classic immune response-related pathways, such as the ERK3, ERK1 + 2, NF-κB p65, and p-STAT3 signaling pathways. The results showed that the protein expression of ERK3 was significantly upregulated in the MPP2 KO ARPE19 cells with the oeMPP2-N315 mutation compared to those with oeWT (Fig. 4E, F). In vivo, ERK3 was significantly increased in the retinas of the MPP2-N315 AAV-transfected mice with EAU (Figs. 4G, I). Moreover, homozygous or heterozygous MPP2-N315 knock-in mice with EAU showed enhanced protein levels of ERK3 (Fig. 4H, J). Previous studies have reported that IL-17E is mainly released by epithelial cells and mediates the epithelium-related inflammatory response [48]. Here, we found that the release of IL-17E was significantly increased in oeMPP2-N315 mutant ARPE19 cells compared to oeWT ARPE19 cells through ELISAs (Fig. 4K). Similarly, in MPP2-N315 AAV mutant or MPP2-N315 knock-in mice with EAU, we also observed higher levels of IL-17E (Fig. 4L, M). In general, our data indicate that MPP2-N315 mutation worsens EAU inflammation through the ERK3/IL-17E pathway.

### Inhibition of ANXA2 in MPP2 knock-in mice attenuates EAU inflammatory phenotypes

**Fig. 5:**
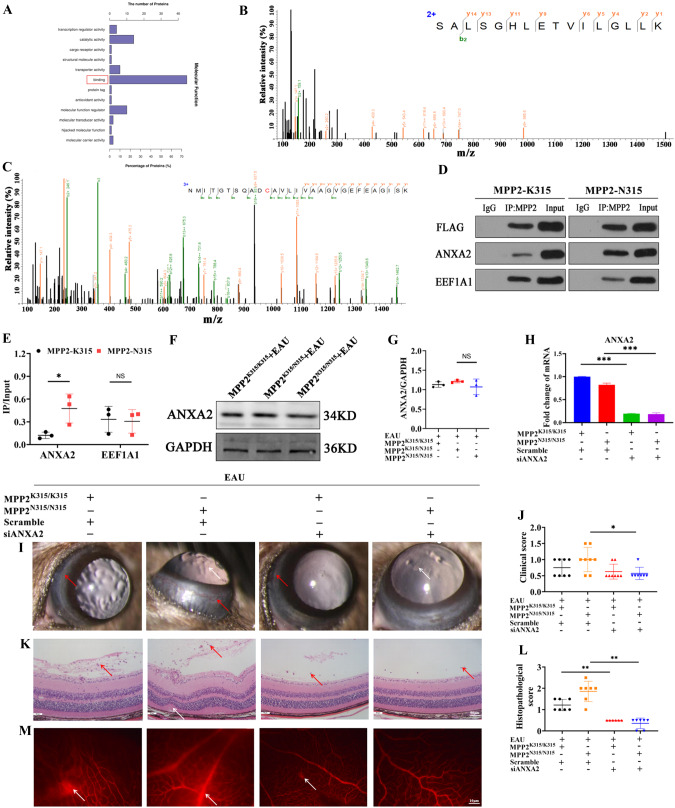
Alleviated EAU inflammation after ANXA2 silencing. **A** Molecular function of MPP2 mutant in proteomics. **B** The top Ion score of the secondary spectrum of ANXA2. **C** The top Ion score of the secondary spectrum of EEF1A1. **D** Co-IP results of MPP2 to ANXA2 or EEF1A1. **E** The grayscale statistics of upper Co-IP results (*n* = 3/group; mean ± SD; NS *p* > 0.05, *p < 0.05; unpaired Student’s *t* test). **F** The protein level of ANXA2 in MPP2-K315 or homozygous or heterozygous MPP2-N315 knock-in mice with EAU. **G** Quantification of ANXA2 in the three groups mentioned above (*n* = 3/group; mean ± SD; NS > 0.05; one-way ANOVA). **H** Knockdown efficiency of ANXA2 in MPP2-K315 or MPP2-N315 knock-in mice with siANXA2 (*n* = 3/group; mean ± SD; ***p < 0.001; one-way ANOVA). **I**, **J** Clinical scores of MPP2-K315 or MPP2-N315 knock-in mice with or without ANXA2 silencing under immunization. White arrow, inflammatory cells. Red arrow, conjunctival and ciliary hyperemia (*n* = 8/group; mean ± SD; *p < 0.05; Mann–Whitney U test). **K**, **L** Histopathological scores in the four groups mentioned above. Red arrow, inflammatory cells. White arrow, retina fold (scale bar, 50 μm.) (*n* = 6–7/group; mean ± SD; **p < 0.01; Mann–Whitney U test). **M** Representative images of Evans blue in the above groups. White arrow, vessel leakage (scale bar, 10 μm)

To further explore how MPP2 mutation mediates the activation of the ERK3/IL-17E pathway, we analyzed the molecular function of MPP2 mutation in proteomics data. We found that the most critical molecular function of MPP2 was its binding with other proteins (Fig. 5A). Consequently, liquid chromatography tandem mass spectrometry (LC‒MS/MS) was performed to detect the ability of MPP2 to bind to other proteins in vitro. The results showed that ANXA2 (sequence: SALSGHLETVILGLLK) and EEF1A1 (sequence: NMITGTSQADCAVLIVAAGVGEFEAGISK) bound directly to MPP2 (Figs. 5B, C). Other lower ion scores of protein secondary spectra for ANXA2 and EEF1A1 are shown in the supplementary information (Supplementary Fig. 4A–C). This interaction relationship was confirmed by Co-IP, and the results showed that ANXA2 has a greater ability to bind with MPP2-N315 than with MPP2-K315 (Fig. 5D, E). Next, we investigated whether MPP2-N315 regulates the expression of ANXA2, and western blotting showed that the MPP2-N315 mutation did not affect the protein level of ANXA2 (Fig. 5F, G). To clarify the effect of ANXA2 on EAU inflammation, we generated ANXA2 knockdown lentiviruses, and the knockdown efficiency of siANXA2-3 was found to be the highest (Supplementary Fig. 4D). Therefore, MPP2-K315 or MPP-N315 knock-in mice were infected with siANXA2-3 lentivirus by subretinal injection. The RT‒qPCR results showed that the knockdown efficiency of ANXA2 in the retina reached >70% (Fig. 5H). Next, MPP2-K315 or MPP2-N315 knock-in mice with or without siANXA2 were induced to generate an EAU model. Clinical and histopathological scores both confirmed a milder inflammatory response in the MPP2 knock-in mice with ANXA2 knockdown on Day 14 (Fig. 5I–L). Moreover, retinal vascular leakage was attenuated after ANXA2 silencing on Day 14 (Fig. 5M).

### Knockdown of ANXA2 decreases the expression of ERK3 and IL-17E

**Fig. 6:**
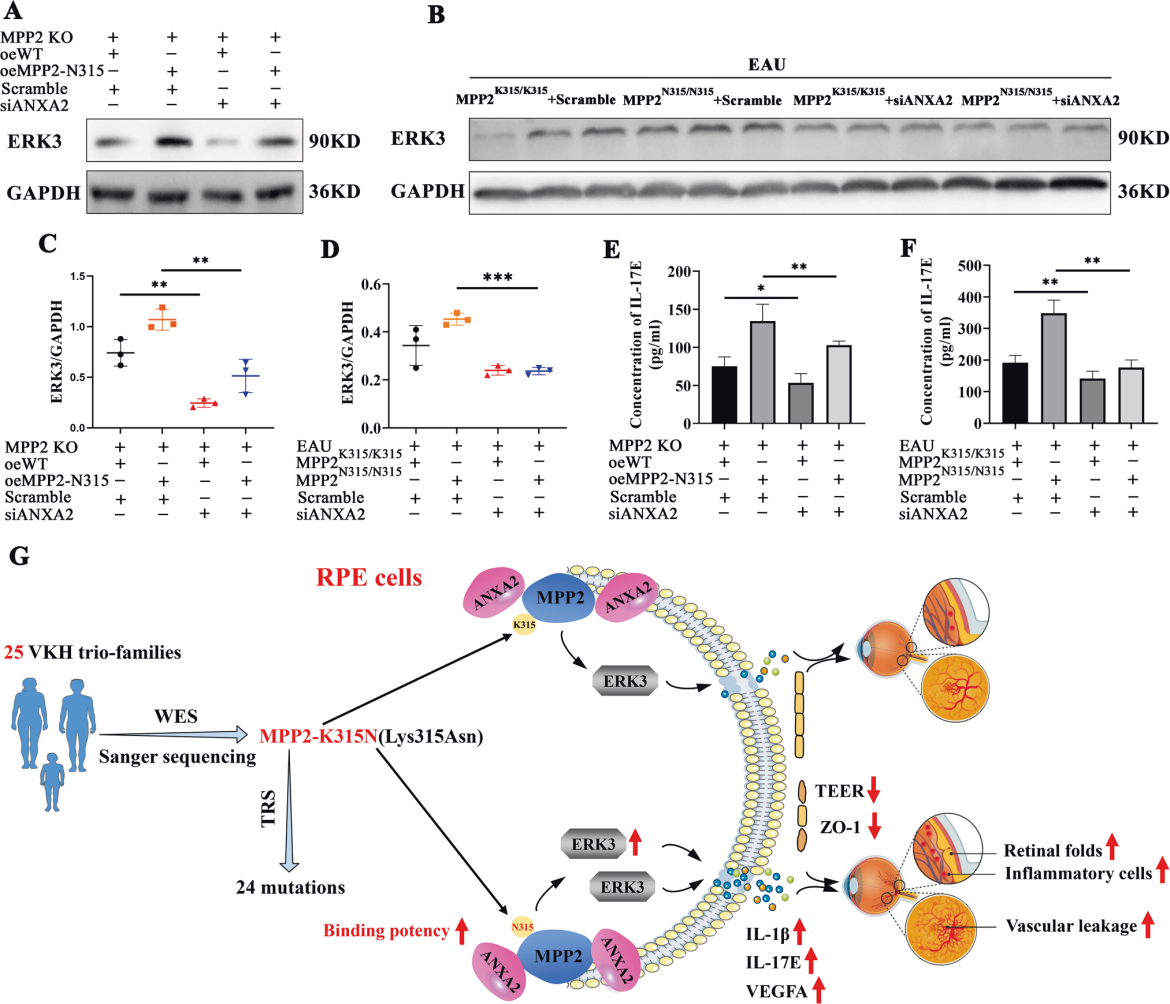
Exacerbation of EAU inflammatory phenotypes via the MPP2/ANXA2/ERK3/IL-17E pathway. **A**, **C** The protein level and quantification of ERK3 in oeWT or oeMPP2-N315 ARPE19 cells with or without siANXA2 based on MPP2 KO (*n* = 3/group; mean ± SD; **p < 0.01; one-way ANOVA). **B**, **D** The expression and quantitative chart of ERK3 in MPP2-K315 or MPP2-N315 knock-in mice with scramble or siANXA2. (*n* = 3/group; mean ± SD; ***p < 0.001; one-way ANOVA). **E** The extracellular protein level of IL-17E (*n* = 6/group; mean ± SD; *p < 0.05, **p < 0.01; one-way ANOVA). **F** The secretion level of IL-17E in vivo (*n* = 6/group; mean ± SD; **p < 0.01; one-way ANOVA). **G** Schematic diagram of the MPP2-N315 mutation exacerbating EAU inflammation through the ANXA2/ERK3/IL-17E pathway

To examine the effect of ANXA2 on the ERK3/IL-17E pathway, we detected the protein expression levels of ERK3 and IL-17E in vitro and in vivo. Based on MPP2 KO ARPE19 cells, ERK3 was reduced in the cells overexpressing WT or the MPP2-N315 mutation with siANXA2 compared to those with scramble (Fig. 6A, C). Similarly, the level of ERK3 was also significantly decreased in the MPP2-K315- or MPP2-N315-knock-in EAU samples with ANXA2 knockdown (Fig. 6B, D). ELISAs showed that IL-17E secretion was significantly reduced both in vitro and in vivo after ANXA2 silencing (Fig. 6E, F). Collectively, these results suggested that the MPP2-N315 mutant can directly bind increased levels of the ANXA2 protein, which then promotes the expression of downstream signaling pathways such as ERK3/IL-17E, resulting in a severe inflammatory response, leakage of retinal vessels, and destruction of the BRB (Fig. 6G).

## Discussion

Prior cumulative studies have indicated that genetic and immune risk factors play important roles in ocular diseases such as age-related macular degeneration, myopia, and uveitis [49–52]. The development of VKH disease is associated with an increased incidence of disease in pigmented populations as well as familial aggregation of disease, and it is strongly related to human leukocyte antigens HLA-DR4 and HLA-DRw53 in specific ethnic populations such as Chinese, Japanese, and other populations [8, 53]. However, the profile of susceptibility to VKH disease remains unclear. In this study, we performed a trio-based WES study including 25 trio VKH patients and a TRS study of 2081 sporadic VKH patients from a Han Chinese population. A total of 15 de novo mutations were identified, with one of the most important and rare being MPP2 p.K315N.

MPP2 is a type of membrane-associated protein belonging to a family of membrane-associated guanylate kinase homologs (MAGUKs). This protein has been demonstrated to be involved in a variety of cellular processes, such as promotion of tight junction formation, polarization of epithelial cells, and apoptosis of hepatocellular carcinoma cells [54, 55]. In addition, this gene was found to act as an exon-specific accessory factor for ribosomal RNA maturation, which affects protein synthesis and controls cell growth by increasing the level of E7 protein [55–57]. MPP2 was reported to be a differentially secreted protein in high glucose–treated ARPE19 cells, and this finding was also validated in plasma from patients with type 2 diabetic retinopathy [58]. A recent study also demonstrated the important role of MPP2 in neurons [59]. The present study identified a de novo mutation, p.K315N, in the MPP2 gene for VKH disease. We then found that this mutation could enhance the release of IL-1β, IL-17E, or VEGFA, which has been reported to worsen the inflammatory phenotype and vascular permeability [46, 60]. Previous studies showed that IL-1β is always linked to pyroptosis; however, in our experiments, we observed an increased level of IL-1β as well as enhanced proliferation in MPP2-transfected ARPE19 cells. These different results may be explained by the fact that IL-1β secreted by RPE cells mainly plays a proinflammatory and proproliferative role in RPE cells rather than mediating the occurrence of pyroptosis mainly in antigen-presenting cells, such as macrophages [19], and then promotes excessive proliferation of ARPE19 cells. In addition, we found that the MPP2-N315 mutation impaired the barrier function of RPE cells, aggravating retinal vascular leakage and inflammatory manifestations in mice with EAU. These results suggest that the MPP2-N315 mutation may play a critical role in maintaining epithelial cell barrier function and initiating the inflammatory response in uveitis.

However, it is still unclear how the MPP2-N315 mutation mediates the activation of inflammatory pathways. To elucidate this mechanism, we performed LC‒MS/MS and found that MPP2 can bind directly to annexin A2 (ANXA2). ANXA2, a member of the calcium-dependent membrane-bound protein family, has been implicated in the development of oncogenesis and various inflammatory diseases, such as hepatocellular carcinoma (HCC), systemic lupus erythematosus (SLE), and inflammatory bowel disease (IBD) [61–63]. In addition, ANXA2 has been shown to act synergistically with its ligand tPA in Pten deletion–induced axon regeneration disease, significantly protecting RGC stomata and preserving visual function in a glaucoma model [64]. Prior studies have demonstrated that ANXA2 upregulates the expression of IL-6, which subsequently activates the ERK pathway and promotes the development of ankylosing spondylitis (AS) [65]. Jung et al. [66] found that ANXA2 increases the transcriptional activity of NF-κB by binding to the p50 subunit of NF-κB. Moreover, NF-κB regulates the expression of IL-17E in various biological contexts [67]. However, the effect of ANXA2 on uveitis has not yet been reported. In this study, we showed the direct binding relationship of ANXA2 and MPP2 using Co-IP. MPP2 and ANXA2 are both membrane proteins. ANXA2 consists of a disordered N-terminal domain (2nd–23rd amino acid) and a highly conserved, rigid C-terminal domain (24–339th amino acid) [68]. A previous study showed that ANXA2^23-239^ could bind directly with the PDZ domain [69]. In particular, MPP2 contains a PDZ motif, as shown in Fig. 1B, and ANXA2 may bind directly with MPP2 through the PDZ domain. Further studies will be performed to elucidate the interaction between these molecules. Furthermore, knockdown of ANXA2 was found to ameliorate the increased EAU severity and recover the barrier function of RPE cells. Mechanistically, ANXA2 aggravated the pathological process of uveitis via the ERK3/IL-17E pathway. Taken together, these results revealed a crucial role of ANXA2 in a variety of autoimmune diseases, such as autoimmune uveitis, SLE, and IBD, via the activation of inflammatory signaling pathways.

The current study has partially elucidated the possible causes of MPP2 de novo mutations in the development of VKH disease. However, some uncertainties remain. In humans, MPP2 consists of 576 amino acids and is expressed in the testis, thyroid, and another 12 tissues; it contains PDZ and SH3 motifs, as shown in Fig. 1B, which are associated with the cytoskeleton and are suspected to play important roles in signal transduction. In mice, MPP2 consists of 552 amino acids and is expressed in the frontal lobe, cortex, retina, and another 20 tissues; it plays a role in cell cycle regulation and signal transduction. In summary, the basic structural and functional properties of human and mouse MPP2 are similar, but there are some differences in the specific expression patterns and molecular mechanisms. More studies are needed to fully understand the differences in MPP2 between the two species.

In addition, although we identified a de novo mutation, MPP2-K315N, where the amino acid lysine at position 315 is replaced with asparagine, the MPP2-K315N mutation is novel and has not been previously reported in human diseases. It is not yet clear whether there is a competitive interaction between MPP2-K315 and MPP2-N315, and further studies will be performed to clarify the interaction between these molecules. In this study, we constructed MPP2-K315N mutation knock-in mice to identify an aggravating ocular disease phenotype. The missense mutation in MPP2 was conserved in 10 species, including humans, monkeys, pandas, rats, and mice. We also detected the expression of MPP2 in a patient with the MPP2-K315N mutation and in MPP2-K315N knock-in mice, and we found that the MPP2-N315 mutation did not affect the gene expression of MPP2. All of these results suggest that the MPP2 missense mutation itself affects the phenotype of the disease. Further studies are needed to elucidate the roles of overexpression of the MPP2-K315N mutant in MPP2-K315N KO mice in the development of uveitis in the future.

During in vitro experiments, we performed a series of functional assays in MPP2 mutant ARPE19 cells without any stimulation, which clarified the effect of MPP2 mutation on the occurrence of uveitis. However, the role of MPP2 mutations in pathological conditions has not been investigated, and further experiments will be carried out on inflammation-stimulated ARPE19 cells with MPP2 mutations to fully elucidate the critical role of MPP2 mutations in the development of uveitis.

In conclusion, we identified a de novo mutation, MPP2 p.K315N (c.G945T, NM_001278372), in VKH disease using WES and then demonstrated the deleterious role of the MPP2-K315N mutation in the occurrence and development of uveitis. Mechanistic studies showed that the MPP2-N315 mutation activates the ERK3/IL-17E pathway by binding directly to more of the ANXA2 protein. Our experiments found that the MPP2-N315 mutation increases the risk of VKH disease, and we uncovered a promising therapeutic target for uveitis.

### Supplementary information

Sup-Fig1-4, Tab1-2

unprocessed original images
