# Supplementary material for: A de novo missense mutation in MPP2 confers an increased risk of Vogt–Koyanagi–Harada disease as shown by trio-based whole-exome sequencing
Source: Cell Mol Immunol. 2023 Oct 12;20(11):1379–92. doi: 10.1038/s41423-023-01088-9 (PMC10616125; doi:10.1038/s41423-023-01088-9)

Fig. 1

MPP2

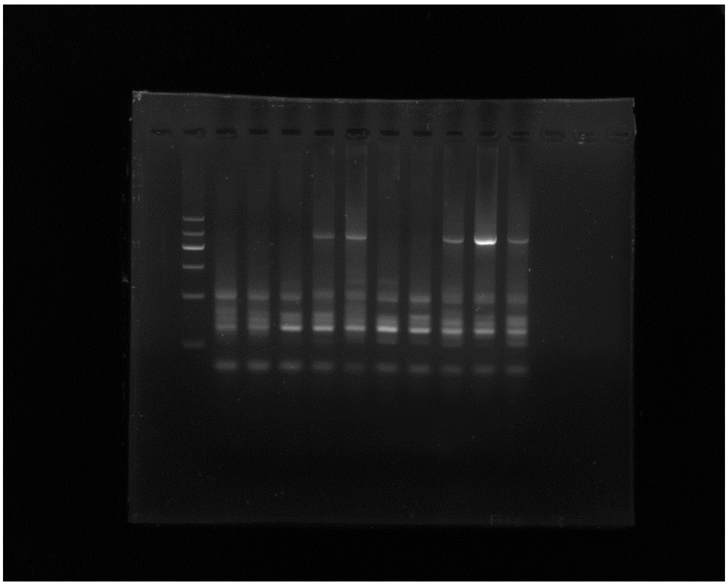

β-actin

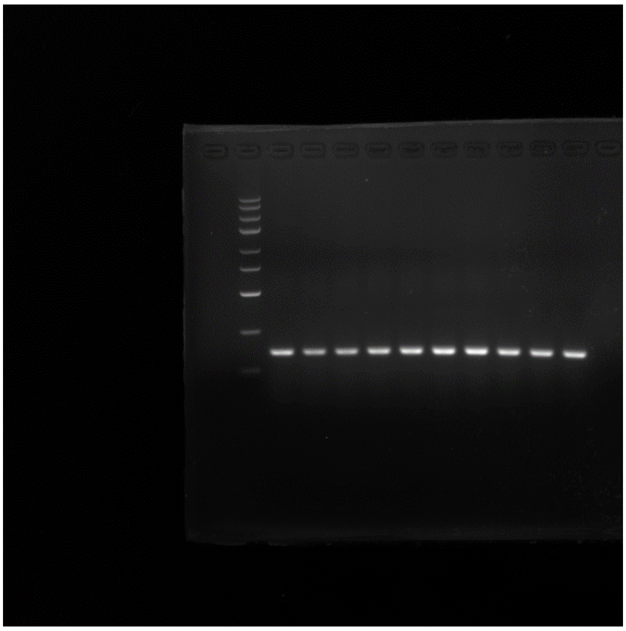

Fig. 3

a

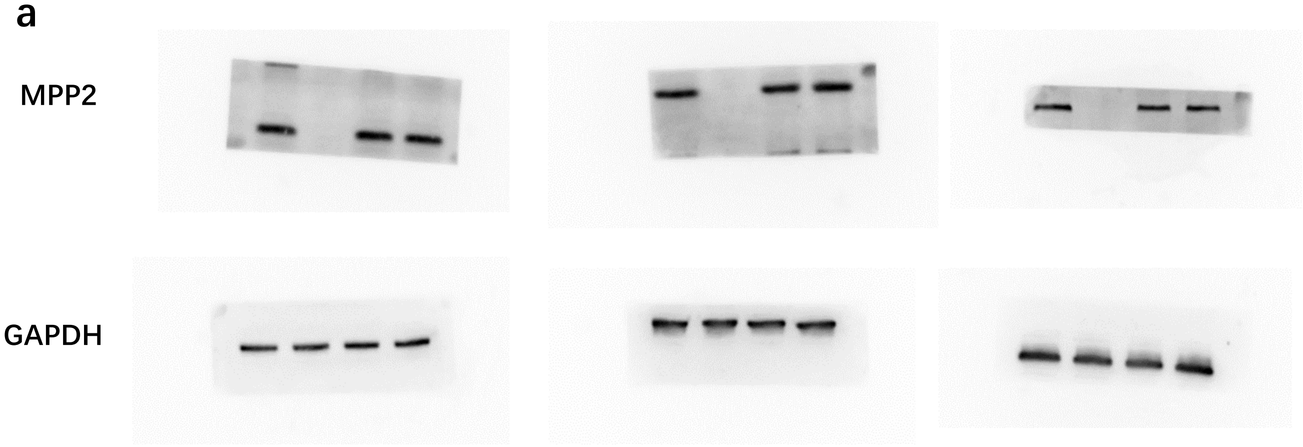

k

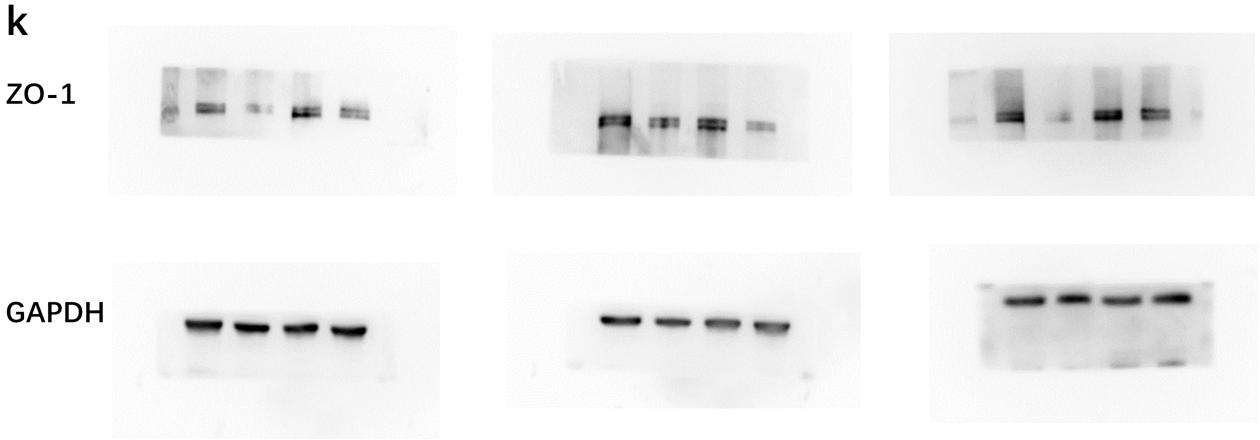

Fig. 4

e

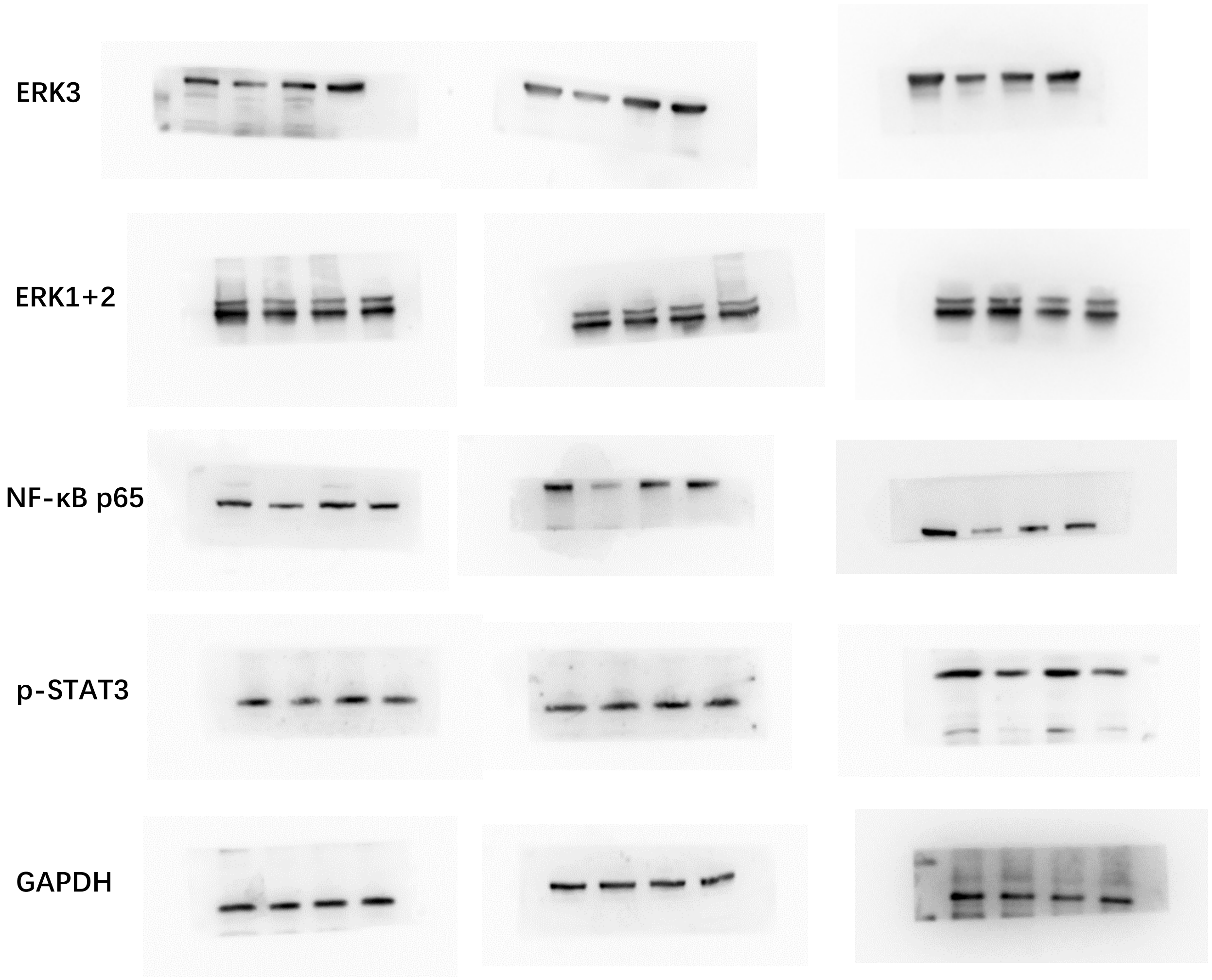

g

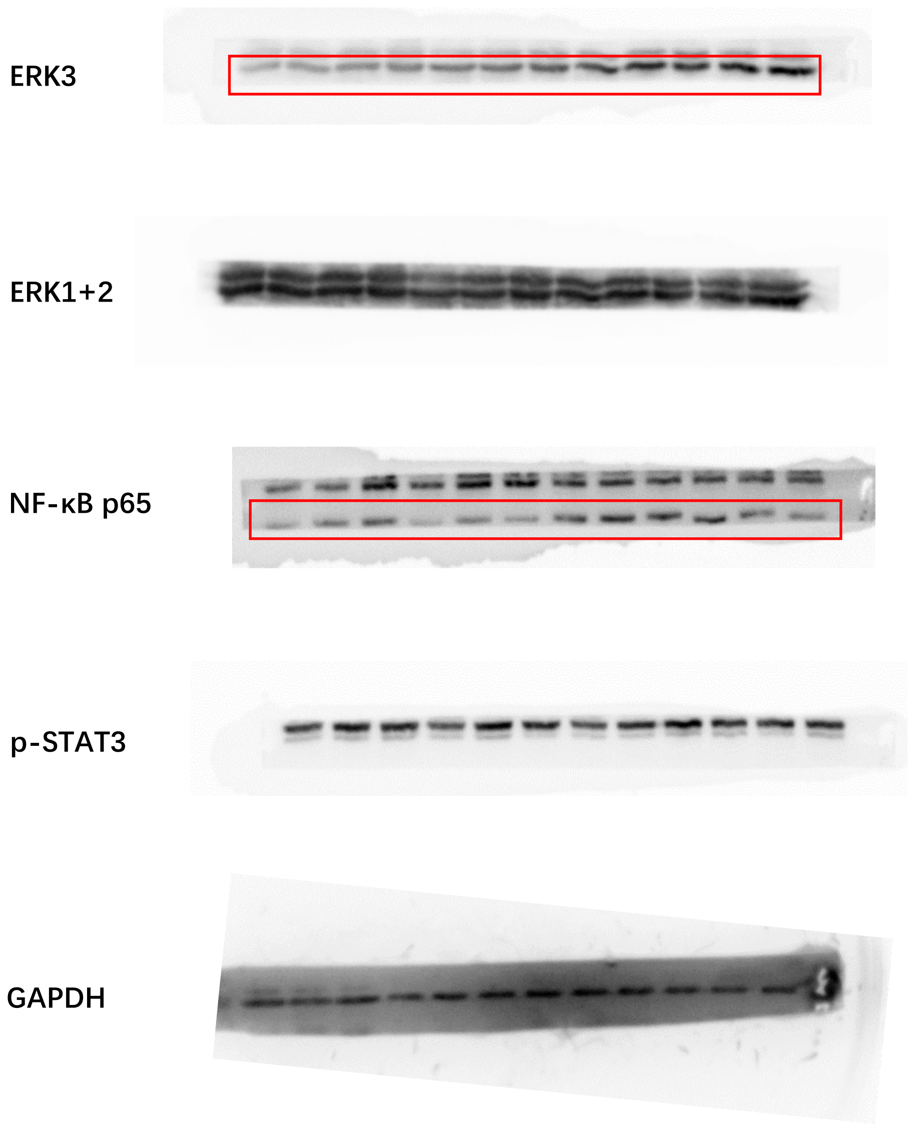

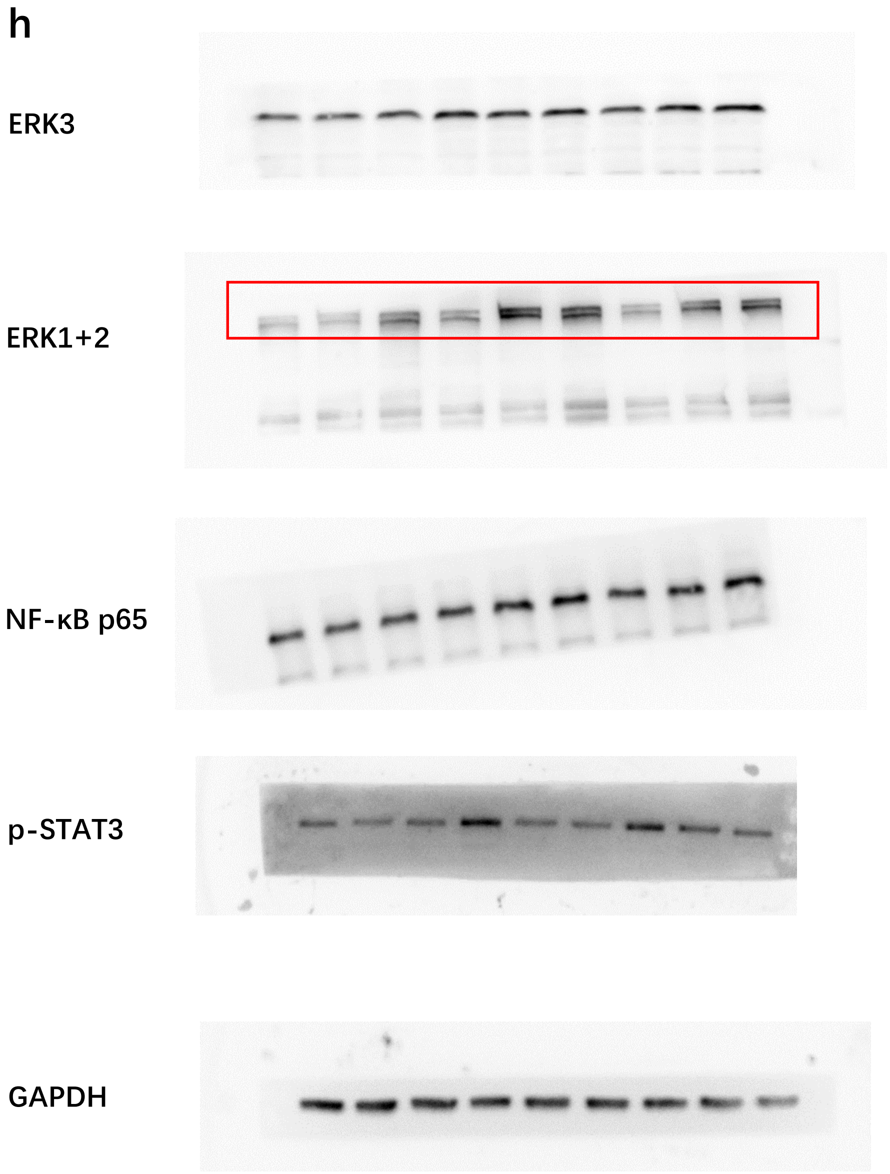
h
ERK3
ERK1+2
NF-κB p65
p-STAT3
GAPDH

Fig. 5

d

MPP2-K315

Flag

ANXA2

EEF1A1

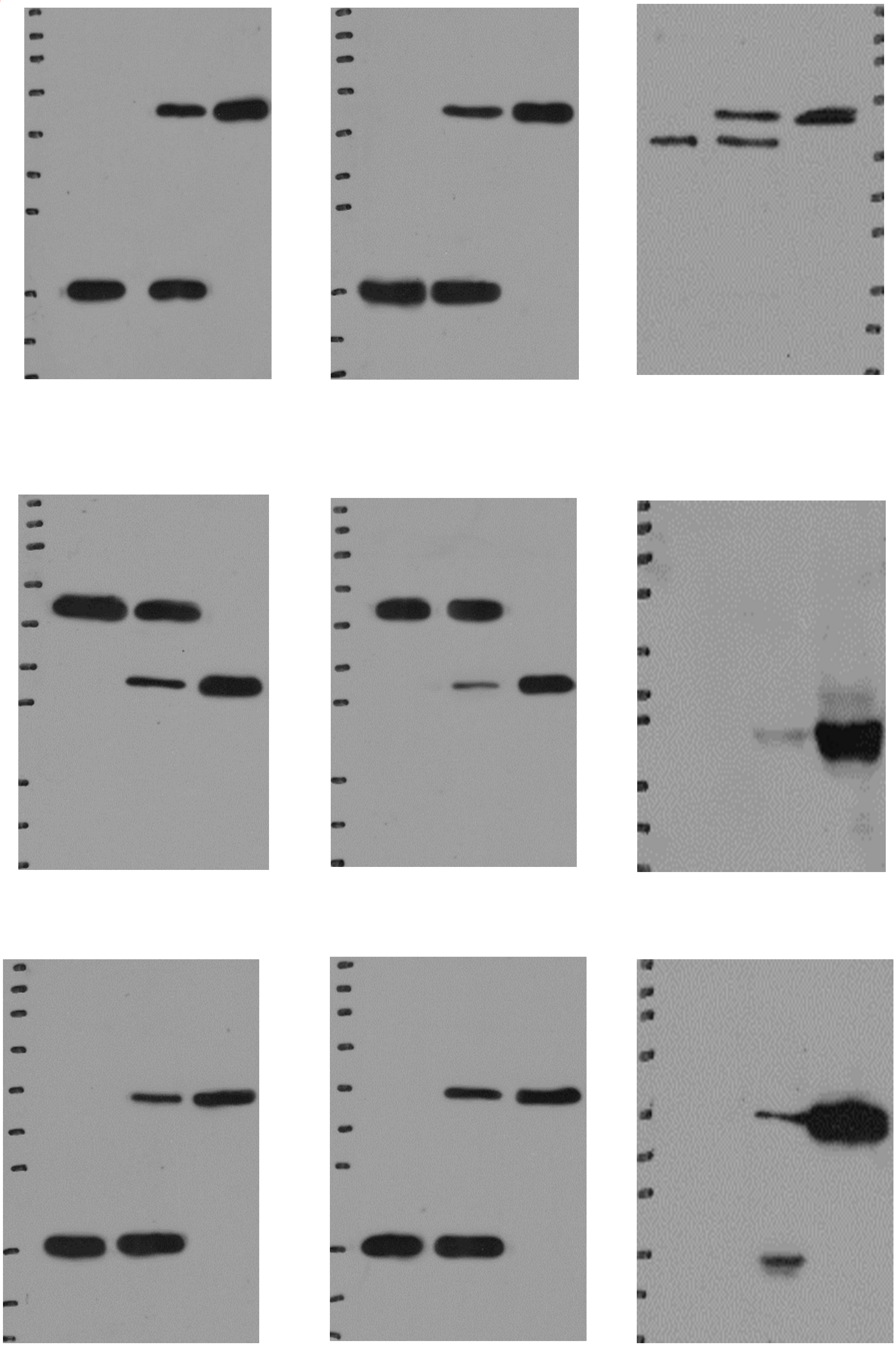

MPP2-N315

Flag

ANXA2

EEF1A1

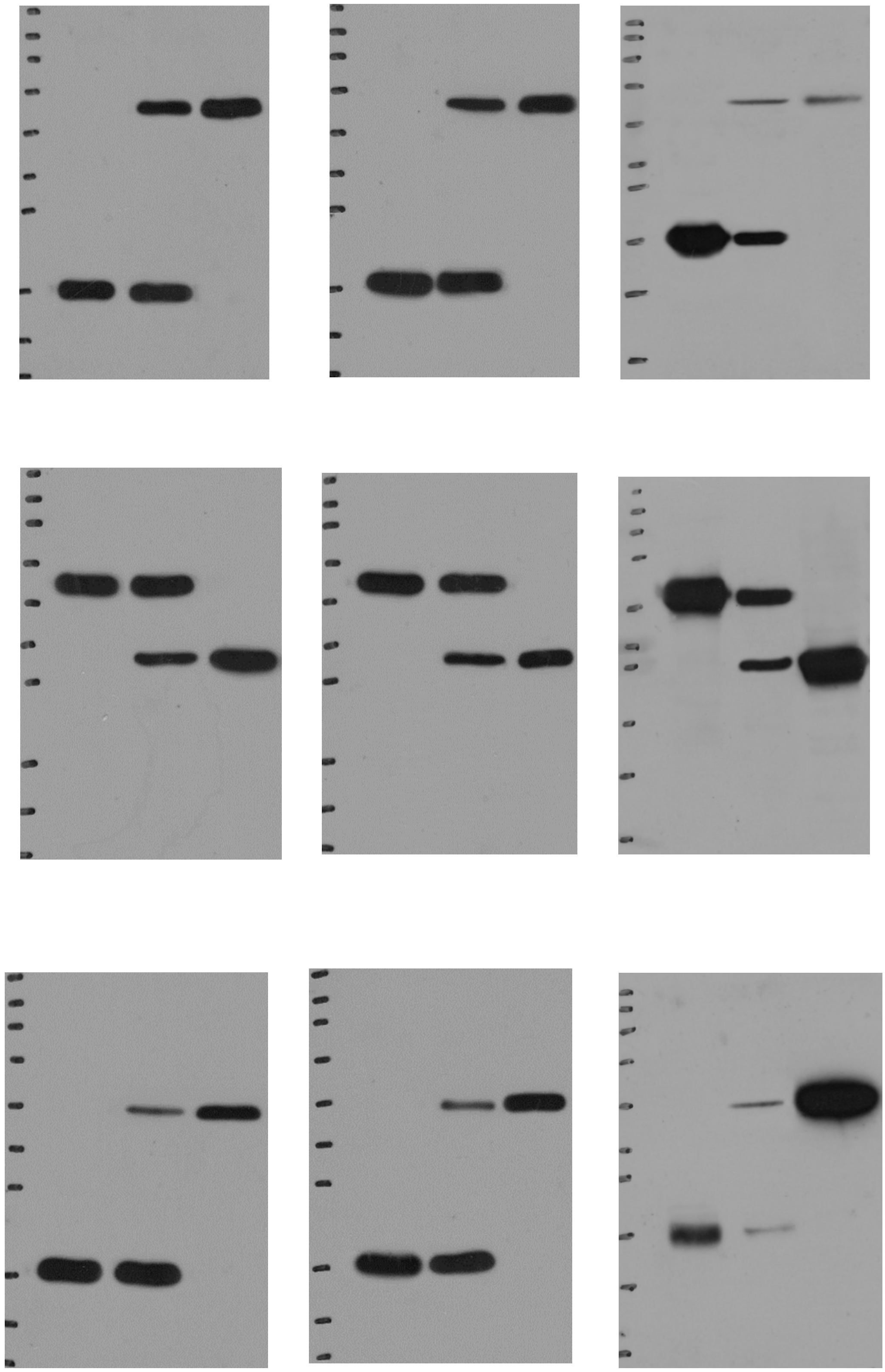

f

ANXA2

GAPDH

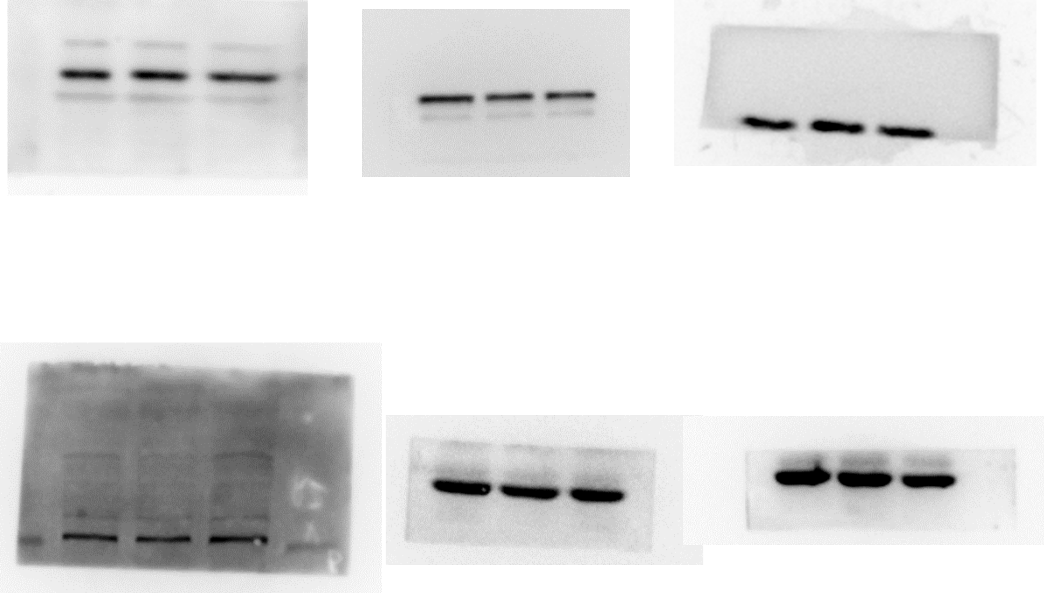

Fig 6

a

ERK3

GAPDH

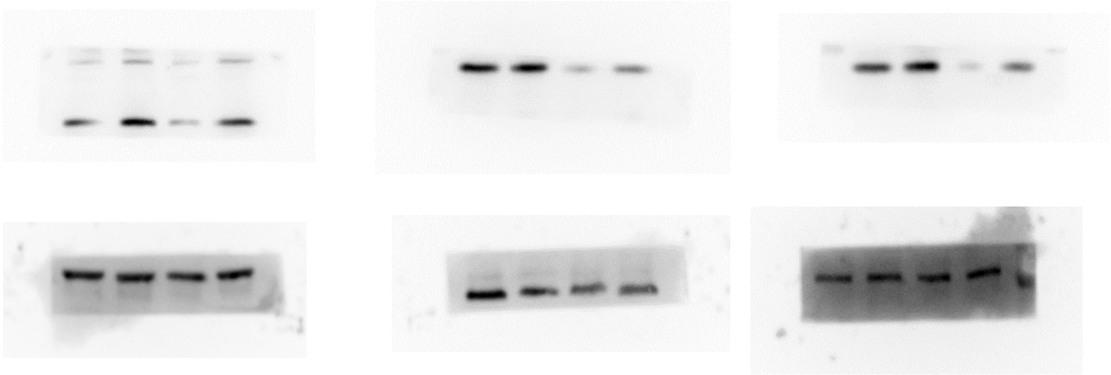

b

ERK3

GAPDH

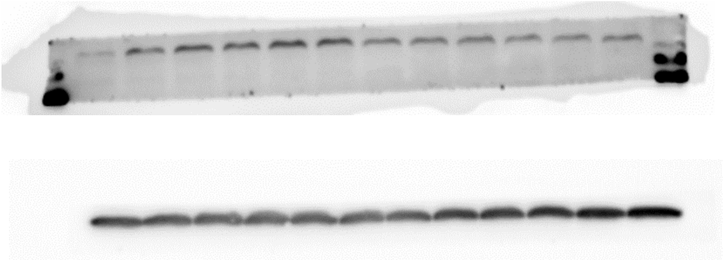

Supplementary Fig. 2

d

EGFP

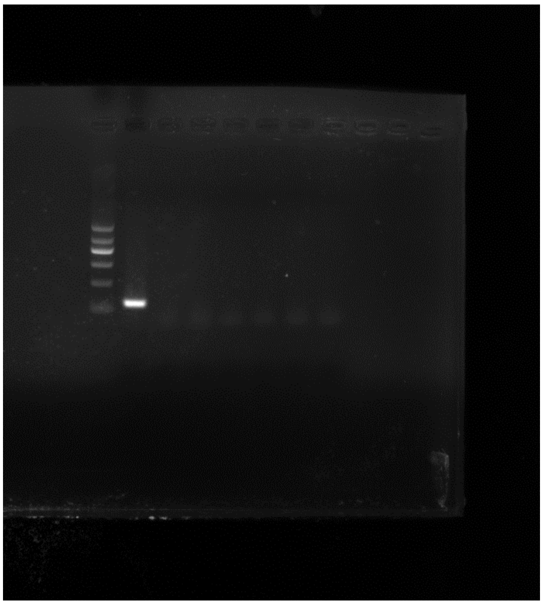

GAPDH

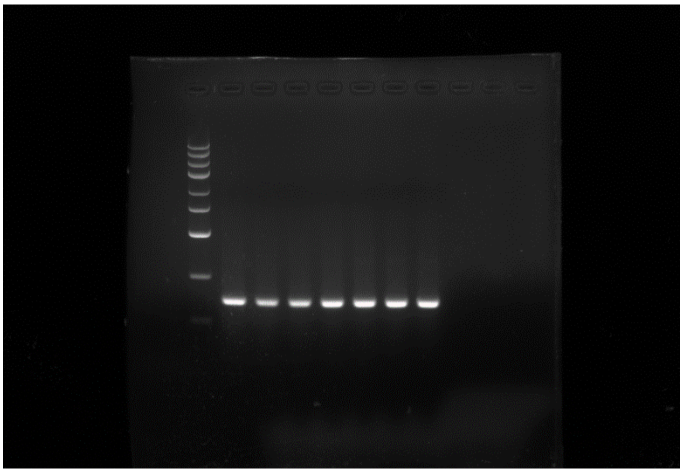

o

MPP2

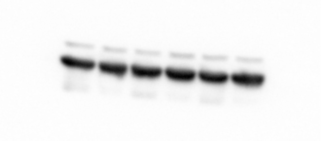

GAPDH

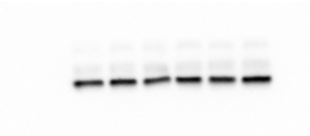

Supplement: Supplementary file 2 — unprocessed original images [file 41423_2023_1088_MOESM2_ESM.pdf]
