# Supplementary material for: A de novo missense mutation in MPP2 confers an increased risk of Vogt–Koyanagi–Harada disease as shown by trio-based whole-exome sequencing
Source: Cell Mol Immunol. 2023 Oct 12;20(11):1379–92. doi: 10.1038/s41423-023-01088-9 (PMC10616125; doi:10.1038/s41423-023-01088-9)
Supplement: Supplementary file 1 — Sup-Fig1-4, Tab1-2 [file 41423_2023_1088_MOESM1_ESM.docx]

**Full title: De novo missense mutation in MPP2 confer an increased risk of Vogt–Koyanagi–Harada disease by Trio-based whole exome sequencing**

**Running title: MPP2 increases** **susceptibility of Vogt–Koyanagi–Harada disease**

Xianyang Liu^1,2,3,#^, Jiayu Meng^1,2,3,4,#^, Xingyun Liao^1,2,3,5,#^, Yusen Liu^1,2,3,#^, Qian Zhou^1,2,3^, Zongren Xu^1,2,3^, Shuming Yin^6^, Qingfeng Cao^1,2,3^, Guannan Su^1,2,3^, Siyuan He^1,2,3^, Wanqian Li^1,2,3^, Xiaotang Wang^1,2,3^, Guoqing Wang^1,2,3^, Dali Li^6^, Peizeng Yang^1,2,3,*^ and Shengping Hou^1,2,3,7,*^

^1^The First Affiliated Hospital of Chongqing Medical University, Chongqing, China

^2^Chongqing Key Laboratory of Ophthalmology, Chongqing, China

^3^Chongqing Eye Institute, Chongqing, China

^4^Sichuan Provincial Key Laboratory for Human Disease Gene Study, Sichuan Provincial People's Hospital, University of Electronic Science and Technology of China, Chengdu 611731, China

^5^Department of Medical Oncology, Chongqing University Cancer Hospital, Chongqing, 400030 China

^6^Shanghai Frontiers Science Center of Genome Editing and Cell Therapy, Shanghai Key Laboratory of Regulatory Biology, School of Life Sciences, East China Normal University, Shanghai, 200241, China

^7^Beijing Institute of Ophthalmology, Beijing Tongren Eye Center, Beijing Tongren Hospital, Capital Medical University, Beijing Ophthalmology & Visual Sciences Key Laboratory, Beijing, 100730, China

#These authors contributed equally

*Corresponding author: Professor Shengping Hou Ph.D, The First Affiliated Hospital of Chongqing Medical University, Chongqing 400016, China; Beijing Institute of Ophthalmology, Beijing Tongren Hospital, Capital Medical University, Beijing, 100730, China

E-mail address: sphou828@163.com; Telephone number: 0086-10-58265941

& Professor Peizeng Yang, MD, Ph.D, The First Affiliated Hospital of Chongqing Medical University, Chongqing 400016, China

E-mail address: peizengycmu@126.com; Telephone number: 0086-23-89012851

**Supplementary information**

**Supplementary figure 1. Expression of MPP2 between MPP2-N315 mutant and non-mutant VKH patients.**

**Supplementary figure 2. Subretinal injection of MPP2 mutant AAVs and time course of clinical symptoms in MPP2-N315 mutant mice after EAU.** (a) Process of subretinal injection. (b, c) The fluorescence intensity of green fluorescent protein (Egfp) between uninjected and injected eye. (d) The expression of MPP2 in various human tissues and cell lines. (e,f) The symptoms of anterior segment in mice with pbs or vehicle or MPP2-K315 or MPP2-N315 mutant AAV. Red arrow, conjunctival and ciliary hyperemia (n=4-9/group; mean ± SD; NS P>0.05, * p<0.05; Mann–Whitney U test). (g, h) Hematoxylin-eosin staining and histopathological scores in pbs or vehicle or wildtype or MPP2-N315 mutant mice. Red arrow, inflammatory cells (Scale bar, 50 μm) (n=6-10/group; mean ± SD; * p<0.05; Mann–Whitney U test). (i) Pictures of Evans blue assay in the three groups mentioned above. White arrow, vessel leakage (Scale bar, 10 μm). (j) The phenotypes of skin in MPP2-N315 knockin mice without EAU. (k, l) The symptoms of anterior segment in MPP2-N315 knockin mice with EAU on day 8, 11, 14, 17, 20. Red arrow, conjunctival and ciliary hyperemia (n=4/group; mean ± SD; * p<0.05; Mann–Whitney U test). (m, n) Hematoxylin-eosin staining and histopathological scores in MPP2-N315 knockin mice with EAU on day 8, 11, 14, 17, 20. Red arrow, inflammatory cells. White arrow, retina fold (Scale bar, 50 μm) (n=3/group; mean ± SD; * p<0.05; Mann–Whitney U test). (o, p) The protein level and quantification of MPP2 between MPP2-K315 and MPP2-N315 knockin mice with EAU (n=3/group; mean± SD; NS p>0.05; unpaired Student’s t-test).

**Supplementary figure 3. Deletion of MPP2 in ARPE19 cells.** (a) Sanger sequencing of the genomic DNA of Cas9-treated ARPE19 cells. (b) The analysis of CRISPR Edits (ICE) software.

**Supplementary figure 4. The binding protein to MPP2.** (a) The other protein secondary spectrum on ANXA2 (sequence: QDIAFAYQR). (b, c) The other protein secondary spectrums on EEF1A (sequence: THINIVVIGHVDSGK; NMITGTSQADCAVLIVAAGVGEFEAGISK). (d) The silenced degree of mRNA in siANXA2 lenviruses (n=3/group; mean ± SD; *** p<0.001; one-way ANOVA).

**Supplementary table 1. The information of primers.**

**Supplementary table 2. The information of antibodies.**

**sFig. 1**

**sFig. 2**

**sFig. 3**

**sFig. 4**

**Supplementary table 1 The Primers for RT-qPCR used in this study**

| **Genes** | **Species** | **Sequences (5’-3’)** |
| --- | --- | --- |
| *Egfp* | Mouse | TGCTTCAGCCGCTACCC |
|  |  | AGTTCACCTTGATGCCGTTC |
| *Gapdh* | Mouse | AGGTCGGTGTGAACGGATTTG |
|  |  | TGTAGACCATGTAGTTGAGGTCA |
| MPP2 | Human | GACATCAGCTTTCCTGGCCT |
|  |  | CAGTGGCTAGGGTGTGACTG |
| ANXA2 | Human | GAGCGGGATGCTTTGAACATT |
|  |  | TAGGCGAAGGCAATATCCTGT |
| β-ACTIN | Human | GGATGCAGAAGGAGATCACTG |
|  |  | CGATCCACACGGAGTACTTG |

**Supplementary table 2 The Antibodies for western blotting used in this study**

| **Antibody** | **Host** | **Manufacturer** | **Application** |
| --- | --- | --- | --- |
| GAPDH | Mouse | Proteintech | 1:10,000 |
| ZO-1 | Rabbit | Proteintech | 1:1,000 |
| ERK3 | Rabbit | abcam | 1:1,000 |
| ERK1/2 | Rabbit | abcam | 1:5,000 |
| NF-KB p65 | Rabbit | abcam | 1:1,000 |
| p-STAT3 | Rabbit | abcam | 1:1,000 |
| IFN-γ | Rabbit | Bioss | 1:200 |
| Flag | Mouse | Origene | 1:1,000 |
| ANXA2 | Rabbit | abcam | 1:5,000 |
| MPP2 | Rabbit | Invitrogen | 1:1000 |
